# Elastic Resistance Bands in Sports Medicine and Rehabilitation: A Narrative Review of Neuromuscular Mechanisms, Prescription, and Functional Applications

**DOI:** 10.3390/jcm15187086

**Published:** 2026-09-12

**Authors:** Eduardo Guzmán-Muñoz, Exal Garcia-Carrillo, Antonio Castillo-Paredes, Felipe Montalva-Valenzuela, Iván Molina-Márquez, Jose Jairo Narrea Vargas, Rodrigo Villaseca-Vicuña, Emilio Jofré-Saldía, Rodrigo Yáñez-Sepúlveda, Dario Barrera-González

**Affiliations:** 1 School of Kinesiology, Faculty of Health, Universidad Santo Tomás, Talca 3460000, Chile; eguzmanm@santotomas.cl; 2 Department of Physical Activity Sciences, Faculty of Education Sciences, Universidad Católica del Maule, Talca 3480112, Chile; exal.garcia@gmail.com; 3 Department of Physical Activity Sciences, Universidad de Los Lagos, Osorno 5290000, Chile; 4 Grupo AFySE, Investigación en Actividad Física y Salud Escolar, Escuela de Pedagogía en Educación Física, Facultad de Educación, Universidad de Las Américas, Santiago 8370040, Chile; acastillop85@gmail.com; 5 Escuela de Entrenador en Actividad Física y Deporte, Facultad de Ciencias Humanas, Universidad Bernardo O’Higgins, Santiago 8370993, Chile; 6 Pedagogy in Physical Education, Faculty of Education, Universidad Adventista de Chile, Chillán 3780000, Chile; ivanmolina@unach.cl; 7 Doctoral Programme in Physical Activity Sciences, Universidad Católica del Maule, Talca 3460000, Chile; 8 Grupo de Investigación en Nutrición, Metabolismo y Ejercicio, Facultad de Ciencias de la Salud, Carrera de Nutrición y Dietética, Universidad Científica del Sur, Lima 15067, Peru; jnarrea@cientifica.edu.pe; 9 School of Educational Sciences and Technology, Physical Education Pedagogy, Faculty of Education, Universidad Católica Silva Henríquez, Santiago 8280354, Chile; rvillaseca@ucsh.cl; 10 Escuela de Ciencias de la Actividad Física, Facultad de Ciencias de la Rehabilitación y Calidad de Vida, Universidad San Sebastián, Santiago 8370040, Chile; emiliojofresaldia@gmail.com; 11 Faculty of Education and Humanities, School of Sports Sciences, Universidad Andres Bello, Viña del Mar 2200055, Chile; rodrigo.yanez.s@unab.cl; 12 School of Medicine, Universidad Espíritu Santo, Samborondón 092301, Ecuador; 13 School of Kinesiology, Faculty of Health Sciences, Universidad Autónoma de Chile, Talca 3460000, Chile

**Keywords:** elastic resistance, resistance bands, sports medicine, rehabilitation, neuromuscular activation, exercise prescription, load monitoring, functional performance

## Abstract

**Background/Objectives**: Elastic resistance bands are widely used in rehabilitation, injury prevention, and athletic conditioning because they are portable, inexpensive, and adaptable to multiple movement planes. However, their mechanical behavior and training dose are often insufficiently characterized. This structured narrative review aimed to synthesize current evidence on the neuromuscular mechanisms, prescription principles, and functional applications of elastic resistance bands in sports medicine and rehabilitation. **Methods**: PubMed, Scopus, Web of Science, and ScienceDirect were searched from inception to 31 May 2026. Peer-reviewed human studies and reviews were included when they addressed mechanical, neuromuscular, biomechanical, prescriptive, rehabilitative, injury-prevention, or performance-related aspects of elastic resistance exercise. Evidence was synthesized narratively to link mechanical configuration, neuromuscular responses, exercise prescription, and functional outcomes. **Results**: Elastic bands provide an ascending, position- and configuration-dependent resistance profile determined by band properties and exercise configuration. When effort and exercise configuration are controlled, elastic resistance can elicit muscle activation and strength adaptations comparable to conventional resistance, while supporting improvements in balance, functional capacity, power, sprint performance, change-of-direction ability, and selected sport-specific outcomes. Its directional versatility and capacity for gradual load adjustment support applications from early rehabilitation to return-to-sport and performance training. Nevertheless, prescription based on band color alone is inadequate, and inconsistent reporting limits reproducibility and study comparisons. **Conclusions**: Elastic resistance is a scalable and clinically relevant loading modality rather than an inherently low-load alternative. Evidence is more established for improvements in muscular strength and functional performance than for direct reductions in injury incidence or successful return-to-sport outcomes. Its effectiveness depends on explicit load quantification, individualized progression, appropriate exercise configuration, and transparent reporting of intervention parameters.

## 1. Introduction

Resistance exercise is widely recognized as a cornerstone of physical health, musculoskeletal rehabilitation, injury prevention, and athletic development [1,2]. A substantial body of evidence links progressive resistance training to improvements in muscular strength and power, bone and metabolic health, body composition, physical function, and sport performance across the lifespan, supporting the view that strength training represents a key component of exercise-based interventions [3,4]. Within sports medicine and rehabilitation, the question is no longer whether resistance should be applied, but how it can be delivered in ways that are effective, individualized, safe, and feasible across the diverse settings in which people train and recover, from elite sport environments to clinical rehabilitation facilities, community programs, and private homes.

Elastic resistance devices, commonly referred to as elastic resistance bands, resistance bands, elastic bands, or elastic tubing, occupy a distinctive position within this landscape. Initially adopted in rehabilitation settings as an accessible means of loading weakened or recovering muscles, they are now widely used in physiotherapy, injury prevention programs, strength and conditioning, gerontology, and high-performance sport [5,6,7]. Their appeal lies in a combination of attributes that few other resistance modalities share: they are inexpensive, lightweight, portable, and easy to store; they can be used in standing, seated, or lying positions and across multiple planes of movement; and they provide comparatively low resistance at the beginning of a movement while progressively increasing tension as the band is stretched [5,7]. These characteristics make elastic resistance especially attractive for populations and settings in which conventional free weights or machines may be impractical, unavailable, poorly tolerated, or difficult to integrate into rehabilitation and return-to-sport routines.

However, elastic resistance should not be understood merely as a low-cost substitute for conventional loading. Unlike free weights or machines, elastic resistance bands generate a variable resistance profile that depends on the mechanical properties of the band and its configuration within the exercise [8,9]. This mechanical signature may influence joint torque, muscle activation, movement control, proprioceptive demands, and the distribution of effort across the range of motion [10,11,12]. Mechanistic and applied studies suggest that elastic resistance can produce meaningful neuromuscular and functional adaptations, including acute responses, such as changes in muscle activation, and chronic adaptations, such as improvements in strength, balance, mobility, and sport-specific performance [6,13,14,15]. However, these findings remain difficult to compare across studies because elastic resistance is often prescribed and reported using inconsistent parameters, with limited information on load-defining configuration variables, progression criteria, and load monitoring.

From a physiological perspective, elastic resistance exercise can elicit relevant acute neuromuscular, cardiovascular, metabolic, and perceptual responses. As resistance and effort increase, greater motor-unit recruitment and muscle activation are required to generate and control force throughout the range of motion. Depending on the exercise configuration, intensity, active muscle mass, and duration, elastic resistance exercise can also increase heart rate, oxygen uptake, ventilation, blood lactate concentration, and perceived exertion, demonstrating that this modality can impose a meaningful systemic as well as local muscular stimulus [14,16,17]. Importantly, these responses are not determined by the band itself, but by the interaction between framings. Thus, the physiological demand of elastic resistance exercise can vary substantially according to how the exercise is configured and prescribed.

Despite their widespread use in clinical and sport settings, the scientific understanding of elastic resistance remains fragmented. Evidence is dispersed across disciplinary silos, including biomechanics, neurophysiology, clinical rehabilitation, gerontology, exercise physiology, and sport science, each of which tends to frame elastic resistance through different concepts, outcomes, and terminology. Moreover, a recurrent practical challenge remains the lack of standardized methods for quantifying and comparing the load across bands, exercises, and studies. This issue limits reproducibility, complicates comparisons across studies, and reduces the precision with which elastic resistance can be implemented for injury prevention, functional recovery, load management, and performance enhancement [18,19]. Importantly, the strength of evidence differs across outcomes, with more established support for improvements in muscular strength and functional performance than for direct reductions in injury incidence or successful return-to-sport outcomes.

Therefore, the purpose of this narrative review is to synthesize and integrate current evidence on the use of elastic resistance bands in sports medicine and rehabilitation. Specifically, this review examines the mechanical and neuromuscular foundations of elastic resistance, the methods used to quantify and progress training load, and their implications for rehabilitation and functional performance. Accordingly, this review organizes current knowledge within a framework linking band configuration, external load, internal response, accumulated training exposure, and functional adaptation.

## 2. Materials and Methods

This study was conducted as a narrative review supported by a structured literature search, predefined eligibility criteria, sequential screening, and thematic evidence synthesis. In this context, structured refers to the use of explicit search sources, predefined eligibility criteria, title/abstract and full-text screening, and an organized framework for evidence synthesis; it does not imply that the review was designed or conducted as a systematic review. The objective of the review was not to estimate pooled quantitative effects but rather to provide an integrative framework that organizes current knowledge regarding the mechanisms underlying elastic resistance, its exercise prescription and load monitoring approaches, and its implications for injury prevention, rehabilitation, functional recovery, and performance across clinical and athletic populations. Accordingly, the review was intended to provide a structured narrative synthesis of relevant evidence identified through the literature search rather than an exhaustive systematic synthesis of all eligible studies.

The literature search was performed using four major electronic databases: PubMed, Scopus, Web of Science, and ScienceDirect, from inception to 31 May 2026. The search strategy was designed to identify studies addressing the conceptualization, mechanical properties, neuromuscular mechanisms, prescription, and application of elastic resistance band exercise in humans. The search combined terms referring to elastic resistance devices with terms related to mechanical and neuromuscular responses, exercise prescription, rehabilitation, injury prevention, and physical or sports performance. Search terms included keywords such as “elastic resistance”, “elastic resistance bands”, “elastic bands”, “resistance bands”, “elastic tubing”, “Thera-Band”, “variable resistance”, “muscle activation”, “electromyography”, “biomechanics”, “joint torque”, “exercise prescription”, “load monitoring”, “load progression”, “injury prevention”, “rehabilitation”, “return to sport”, “functional recovery”, “functional performance”, and “sports performance”. Boolean operators (AND and OR) were used to combine the terms, and the search syntax was adapted to the requirements of each database. In addition to the database search, the reference lists of relevant articles and reviews were manually screened in order to identify additional publications that could contribute to the conceptual, methodological, and applied framework of the review.

Studies were considered eligible if they addressed elastic resistance bands and examined mechanical, methodological, neuromuscular, biomechanical, prescriptive, rehabilitative, injury-prevention, or performance-related aspects. Human studies were included for physiological, neuromuscular, clinical, rehabilitation, and performance outcomes, while non-human experimental studies were also considered when their primary purpose was the mechanical characterization, force–elongation behavior, or load quantification of elastic resistance devices. Both original research articles and review papers published in peer-reviewed scientific journals were considered when they contributed to the theoretical understanding, methodological approaches, or functional implications of elastic resistance band exercise. Studies focusing exclusively on animal models, those unrelated to elastic resistance exercise, conference abstracts, protocols, letters to the editor, or publications not available in English or Spanish were excluded. Titles and abstracts identified through the search process were initially screened to determine potential relevance, after which full texts of the selected articles were reviewed to confirm their eligibility.

The selected literature was analyzed with the aim of identifying recurring conceptual themes and methodological approaches related to elastic resistance band exercise. Particular attention was given to the definition and mechanical characteristics of elastic resistance bands, the neuromuscular and biomechanical responses elicited by elastic resistance, the variables and tools used to prescribe, quantify, monitor, and progress elastic resistance training, and the functional implications of elastic resistance across rehabilitation, injury prevention, and sport performance contexts. Given the heterogeneity of the literature, different study designs were used according to the question being addressed. Mechanical, biomechanical, and electromyographic studies were primarily used to inform mechanistic interpretation, whereas systematic reviews, meta-analyses, and intervention studies were given greater interpretive weight when evaluating clinical, functional, and performance-related outcomes. Population context was also retained during interpretation, and findings derived from healthy or athletic participants were not assumed to be directly transferable to clinical populations unless supported by corresponding clinical evidence. The evidence was synthesized narratively to develop an integrative conceptual framework linking mechanical principles, neuromuscular mechanisms, exercise prescription strategies, and applied implications in sports medicine and rehabilitation.

## 3. Conceptual and Mechanical Framework of Elastic Resistance Bands

### 3.1. Definition and Characteristics of Elastic Resistance Bands

Elastic resistance refers to the external load generated by the deformation of an elastic material, typically natural or synthetic rubber, that resists being stretched and stores elastic potential energy as a function of its elongation [20,21]. In practice, elastic resistance is provided through several device formats, including flat therapeutic bands, tubular cords with or without handles, and continuous-loop bands of varying widths and thicknesses [21]. Within a given product line, manufacturers commonly use successive colors to represent progressively greater stiffness; however, color codes and the corresponding resistance levels are not standardized across manufacturers [8,22]. Although ISO 20957-2:2024 establishes safety requirements for stationary strength-training equipment that may use elastic cords as a means of resistance, it does not standardize the color coding or force–elongation characteristics of stand-alone elastic resistance bands [23]. To our knowledge, no international standard currently harmonizes the relationship between band color and resistance across manufacturers, representing an important gap for load prescription, comparison, and reproducibility.

A defining feature of elastic resistance devices is that the resistance they provide is not fixed but depends on how far the material is stretched [21,24]. Unlike a dumbbell or weight stack, which presents a constant mass regardless of joint angle, an elastic band exerts minimal force when slack and progressively greater force as it lengthens [8,9]. This property produces an ascending band-tension profile as elongation increases; however, the resulting joint load remains position-dependent because it is also influenced by exercise geometry, joint angle, and moment arms [14,25,26]. It also makes elastic devices uniquely portable and scalable: the same band can deliver almost negligible or substantial resistance depending on its initial length, anchoring position, and the range over which it is stretched. This adaptability partly explains their broad adoption across rehabilitation, injury prevention, and athletic settings [27,28].

Historically, elastic resistance entered clinical practice as a low-threshold means of loading weakened or recovering muscles, and its early adoption in physiotherapy established many of the color-coded protocols still in use today. From a mechanical perspective, elastic bands behave less like fixed external loads and more like tunable spring-like systems whose effective resistance is configured by the practitioner through band stiffness, pre-stretch, anchoring, and movement range [5]. During the phase in which the band elongates, the user works against progressively increasing tension; during the shortening phase, the elastic recoil must be actively controlled [29]. The relationship between these phases and the concentric or eccentric muscle actions depends on the exercise configuration.

### 3.2. Elastic Resistance Versus Conventional Resistance

Conventional resistance training relies predominantly on gravity acting on an external or body mass, whether through free weights, selectorized machines, or body-weight exercises [30]. In these modalities, the external load is relatively constant in magnitude, although its mechanical effect varies according to joint angle, limb position, lever length, and movement trajectory [7]. Moreover, gravitational resistance acts vertically downward, which may limit the directions in which load can be applied. Elastic resistance differs in two important ways: the load magnitude increases with elongation rather than remaining constant, and the direction of resistance is determined by the line of pull between the anchor point and the moving body segment [5,21]. This allows resistance to be applied in horizontal, diagonal, and rotational planes that are often difficult to load with free weights alone.

In routine rehabilitation, ankle and wrist cuff weights represent another practical form of external resistance. Like elastic bands, they are inexpensive, portable, and readily applicable in clinical and home-based settings; however, their mechanical behavior differs substantially. Cuff weights provide an external mass that can be quantified directly and generate predominantly gravity-dependent resistance, whereas elastic resistance varies with elongation and can be oriented according to the anchoring configuration [24,31]. Cuff weights may therefore be useful when simple and reproducible quantification of low external loads is prioritized, while elastic resistance may offer advantages when multidirectional loading or smaller, configuration-based adjustments in resistance are required. These modalities should be regarded as complementary rather than universally interchangeable, with selection guided by the therapeutic objective and exercise context.

A central question for practitioners is whether these mechanical differences compromise the training stimulus. Comparative evidence is broadly reassuring. A systematic review and meta-analysis comparing elastic resistance as a loading modality with conventional resistance training found no superiority of either modality for strength gains in the upper or lower limbs, indicating that both can produce comparable strength adaptations when appropriately programmed [32]. Importantly, this modality-based comparison should be distinguished from comparisons of resistance profiles, in which variable-resistance training is contrasted with constant-resistance training. In this regard, a more recent systematic review and meta-analysis reported greater improvements in maximum strength with variable resistance [33]. Acute comparisons of muscle activation similarly show that elastic devices can elicit electromyographic (EMG) activity in prime movers that is not significantly different from that produced by dumbbells, machines, or barbells when effort is matched by perceived exertion or maximal effort [14,34,35,36]. Comparable findings have also been reported in multi-joint exercises, where elastic resistance and conventional equipment can elicit similar activation patterns under matched effort conditions [37]. Taken together, these findings suggest that elastic resistance should not be considered inferior to conventional loading; rather, its effects depend on how the resistance is configured, loaded, and progressed. These findings support the view that elastic resistance is a legitimate loading modality, rather than merely a low-intensity adjunct, for strength training and rehabilitation.

At the same time, elastic and conventional resistance are not mechanically interchangeable. Because elastic load is usually lower at the beginning of the range of motion and higher toward end-range, the within-repetition distribution of force differs from that produced by constant external loads [33]. This has implications for where in the range of motion the muscle, joint, or movement pattern is most challenged [35]. Elastic resistance also differs from free weights in its inertial characteristics and does not naturally impose the same eccentric overload associated with controlling a descending mass, unless this is specifically engineered through the exercise configuration. Therefore, the choice between elastic and conventional resistance should be guided not by an assumption of equivalence, but by the specific mechanical, clinical, or performance demand the practitioner intends to impose.

### 3.3. Variable Resistance and the Force-Elongation Relationship

The hallmark of elastic resistance devices is variable resistance: the external load changes systematically across the range of motion as a function of material elongation [21,38]. Quantitative characterization of this behavior shows that rubber-based bands may follow curvilinear tension–deformation patterns over broader ranges of stretch, although within practical working ranges the force–elongation relationship is often highly predictable and may approximate linear behavior after initial slack is overcome [8,26,39]. Across a practical working range, coefficients of determination frequently exceed 0.95–0.97, supporting the development of prediction equations and reference tables that estimate band tension from elongation and enable more reproducible load prescription [39].

This variable profile is often described as an ascending resistance curve because force increases as the band is stretched toward the end range [38]. When superimposed on the force-generating capacity of the musculoskeletal system, this profile can be used to better match external load to changes in joint mechanical advantage across the movement. In multi-joint barbell exercises such as the squat, bench press, and deadlift, adding elastic bands increases resistance in the later phase of the lift, where athletes are often mechanically stronger, and may reduce the need for deceleration near lockout while increasing the demand for continued force production through the range of motion [38,40,41]. A systematic review and meta-analysis comparing variable- and constant-resistance training concluded that variable resistance, including band-based methods, can produce comparable and, in some contexts, superior gains in maximal strength, potentially because of a more favorable distribution of loading across the range of motion [33]. A more recent systematic review and meta-analysis focused on athletes likewise concluded that variable resistance, including band-based loading, produces meaningful gains in lower-limb explosive power, reinforcing the performance relevance of the ascending load profile [42].

The same property that makes elastic resistance mechanically attractive also complicates load prescription. A single nominal band does not represent a single fixed load. The force experienced by the user depends on initial length or pre-stretch, anchoring position, movement trajectory, and the elongation achieved during the exercise, all of which vary according to limb length, joint range, technique, and exercise set-up. Recognition of this issue has motivated the laboratory quantification of band tension and the development of band- and distributor-specific loading equations. These tools allow practitioners to convert a target force into a prescribed elongation rather than relying solely on band color [8,19,22]. This is particularly relevant in sports medicine and rehabilitation, where precise load progression is essential for tissue tolerance, injury prevention, functional recovery, and performance optimization.

**Figure 1:**
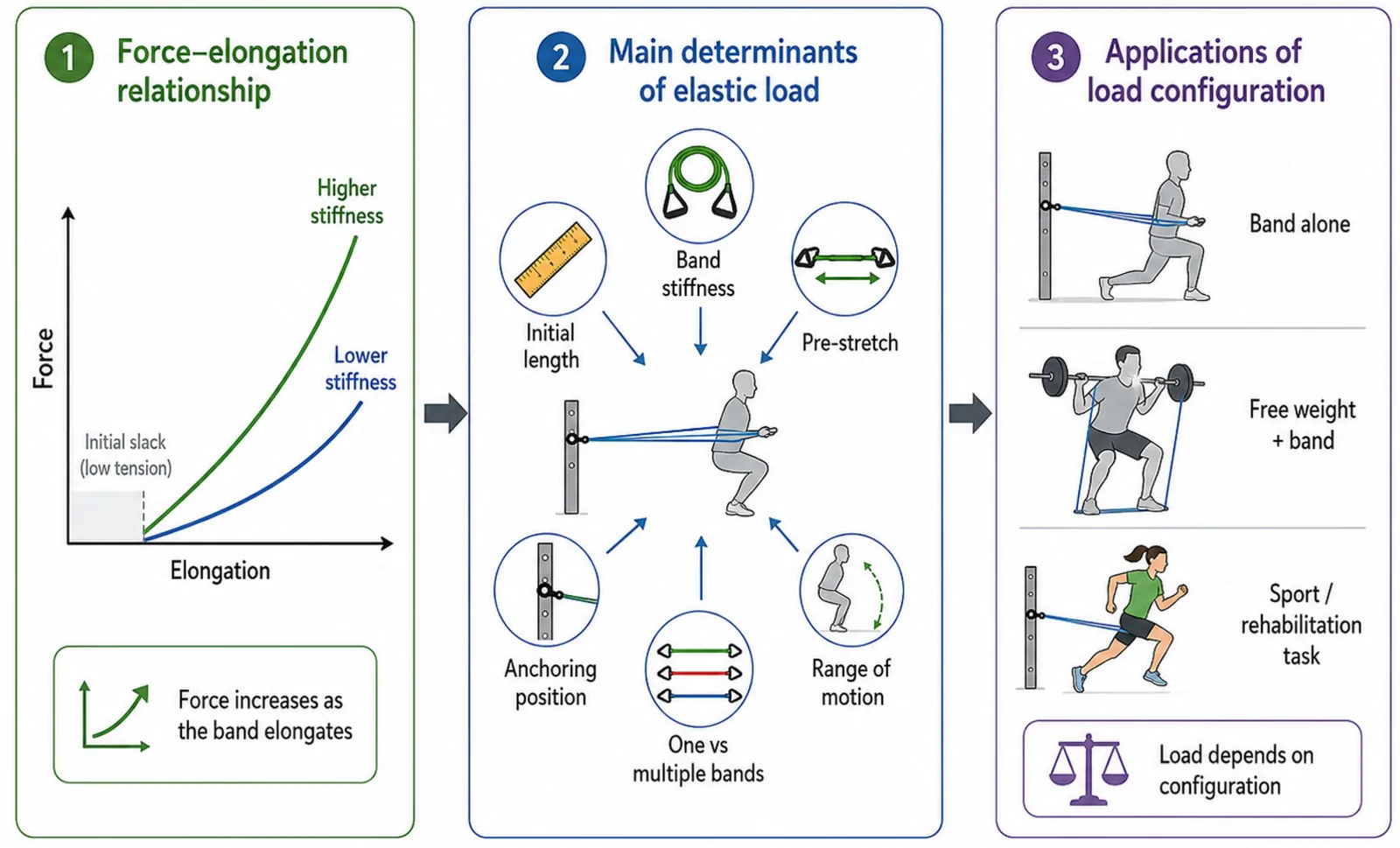
Schematic representation of the force–elongation relationship and the main determinants of elastic resistance loading. The left panel illustrates the ascending force–elongation profile of elastic bands, showing that force increases as elongation increases and differs according to band stiffness. The central panel summarizes the main variables that determine elastic load, including band stiffness, initial length, pre-stretch, anchoring position, range of motion, and the use of one or multiple bands. The right panel presents representative applications of different loading configurations, including stand-alone band exercise, combined free-weight and elastic resistance, and sport- or rehabilitation-specific tasks. The figure emphasizes that the mechanical load imposed by elastic resistance depends on its configuration rather than on band color alone.

Figure 1 illustrates the force–elongation behavior of elastic resistance and summarizes the main variables that determine the load imposed by band-based exercise, as well as representative applications of different loading configurations. Importantly, the force–elongation relationship describes the external tensile force generated by the band and should not be interpreted as a direct measure of joint load or muscle force, which also depend on exercise geometry, moment arms, joint position, and internal musculoskeletal mechanics.

### 3.4. Task, Individual, and Environmental Constraints

The functional behavior of an elastic resistance band is best understood not as a fixed property of the device, but as an emergent outcome of the interaction between the task, the individual, and the environment. This perspective is consistent with constraints-based models of movement coordination, in which motor behavior emerges from the dynamic interaction of task, organismic, and environmental constraints [43]. In the context of elastic resistance exercise, this framework is particularly useful because the load experienced by the user is not determined by band color alone, but by the way the band is configured within a specific movement, person, and setting.

Task constraints include the exercise selected, the plane and direction of movement, the anchoring configuration, the prescribed range of motion, the initial band length, the amount of elongation achieved, and the intended movement velocity. Each of these factors alters how band tension is translated into joint loading, muscle activation, and movement demand [25,44,45]. For example, changing the anchor point may transform the same band from a primarily sagittal-plane resistance into a rotational or diagonal stimulus, whereas increasing pre-stretch or range of motion increases the force generated during the movement [5,25]. Recent biomechanical evidence further supports this configuration-dependent behavior, showing that band stiffness, elongation, and starting length are key determinants of tensile force generation [39]. Therefore, in elastic resistance exercise, the task does not merely determine which movement is performed; it defines the mechanical stimulus imposed on the body.

Individual mechanical characteristics, such as limb-segment length and available range of motion, influence band elongation and the resulting external load. In contrast, strength capacity, pain, tissue tolerance, training status, and rehabilitation stage influence the internal response and determine whether the configured load is appropriate for the individual. These factors determine how much the band is actually elongated during a given exercise and how the resulting tensile force is transferred to the musculoskeletal system. An identically anchored band may therefore impose different relative loads on a tall versus a short individual, on a post-operative patient versus a healthy athlete, or on an early-stage rehabilitation task versus a late-stage performance drill [21]. Importantly, the tensile force generated by the band should not be interpreted as equivalent to the joint torque experienced by the user. The effective mechanical load at the joint also depends on the attachment site, limb position, joint angle, and resulting moment arm [21,39]. This distinction is especially relevant in sports medicine and rehabilitation, where the same external resistance may be therapeutic, insufficient, or excessive depending on tissue irritability, healing stage, and movement tolerance.

Environmental constraints include the available space, anchor points, surface characteristics, equipment access, and degree of supervision. These factors strongly influence whether and how elastic resistance can be deployed across clinical, gym-based, field-based, home-based, and telerehabilitation settings. The portability and low cost of elastic bands make them particularly attractive in environments where conventional machines or free weights are unavailable or impractical. However, reduced supervision may also compromise the consistency of band length, anchoring position, range of motion, and movement execution. Thus, the same accessibility that makes elastic resistance useful in real-world settings also increases the need for clear instructions, standardized set-ups, and simple monitoring strategies to preserve training fidelity.

Framing elastic resistance through this constraints lens highlights the opportunity to deliberately shape the load profile according to task, individual, and environmental constraints [22,26]. By manipulating these configuration-dependent variables, practitioners can tune the resistance stimulus to a specific clinical or performance goal [5,29]. In early rehabilitation, this may involve minimizing load in painful or vulnerable ranges; in later rehabilitation, it may involve progressively increasing tissue tolerance and neuromuscular control; and in return-to-sport or performance settings, it may involve directing resistance toward sport-specific planes, velocities, or movement patterns.

Elastic resistance can also be combined with constant external loads to blend their respective mechanical profiles, as when bands are added to a barbell so that the athlete experiences both a constant gravitational load and an ascending elastic load within the same repetition [34,46]. This represents a deliberate manipulation of task constraints, allowing practitioners to engineer a composite resistance profile that neither modality could produce alone. The ability to layer, anchor, orient, and progress resistance reinforces the central message of this section: in elastic resistance exercise, the load is configured by the user, the task, and the context, rather than dictated by the device alone.

**Figure 2:**
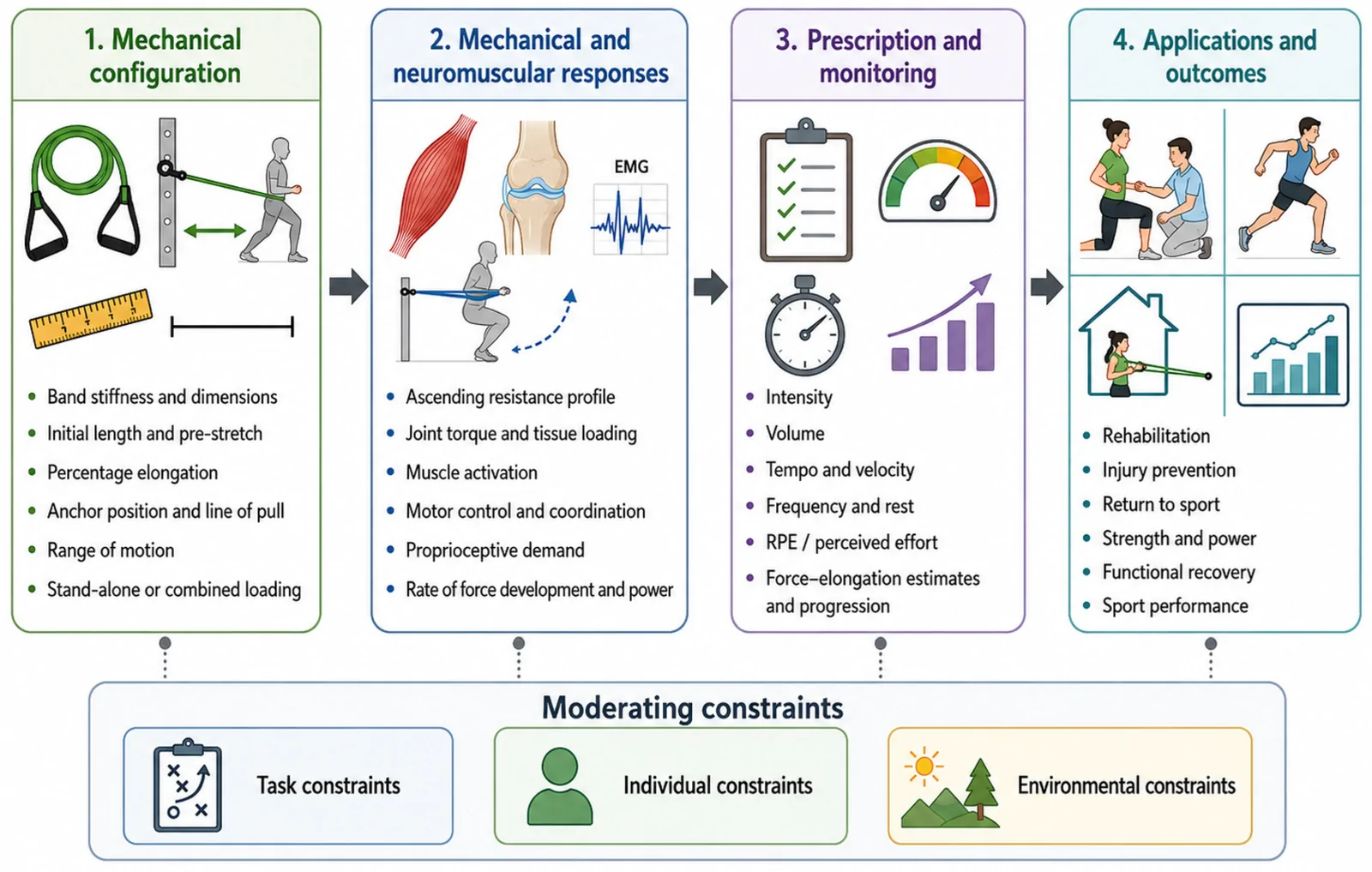
Integrative framework linking the mechanical properties of elastic resistance to neuromuscular responses, exercise prescription, and functional outcomes. The mechanical configuration determines the external load, which interacts with individual characteristics to produce an internal response. Monitoring this response informs subsequent load adjustments, while accumulated training exposure may lead to functional, rehabilitation, and performance adaptations. Task, individual, and environmental constraints moderate each stage of this process and determine the actual load experienced by the user.

Figure 2 summarizes the proposed integrative framework linking the mechanical configuration of elastic resistance to neuromuscular responses, exercise prescription, and functional outcomes. The framework also highlights the moderating influence of task, individual, and environmental constraints on the load experienced and the resulting clinical or performance response. The subsequent sections of this review follow the conceptual sequence summarized in Figure 2, progressing from the mechanical configuration of elastic resistance and the external load imposed, to the resulting neuromuscular responses, training exposure, and functional applications.

## 4. Neuromuscular and Biomechanical Mechanisms

### 4.1. Muscle Activation and Electromyographic Responses

A central concern when elastic devices were proposed as alternatives to conventional resistance modalities was whether their ascending load profile could generate sufficient neuromuscular drive, given that resistance is often lower at the beginning of the range of motion. Comparative surface electromyography (sEMG) studies have provided relevant evidence regarding the capacity of elastic resistance to elicit substantial muscle activation. In single-joint upper-limb exercises, normalized EMG amplitudes of prime movers during elastic resistance are comparable to those produced by dumbbells when effort is appropriately matched, with both modalities capable of eliciting high levels of muscle activation at individually prescribed or maximal loads [34,36]. A meta-analysis pooling available comparisons concluded that, overall, muscle activation does not differ significantly between elastic and isoinertial resistance, supporting elastic devices as a valid means of achieving training-relevant neuromuscular activation [14]. More recent meta-analytical evidence corroborates these findings across the full range of motion, further highlighting that elastic resistance can elicit significantly greater muscle activation during the concentric phase compared to free weights [11]. Additionally, contemporary sEMG analyses demonstrate that while absolute load equivalencies may vary, the neuromuscular synergy and movement symmetry remain highly comparable between both modalities [47].

Closer inspection, nevertheless, reveals modality- and phase-specific differences. Because elastic tension is usually lower early in the movement and greater toward end-range, prime-mover activation may peak later during the resisted phase than with constant external loads [35,36]. Accordingly, machine-based resistance has been shown to elicit greater activation than elastic resistance during some early-range concentric and late-range eccentric portions of contraction, reflecting differences in how the load is distributed across the movement. These phase-specific differences appear to be most evident when the band is relatively slack, particularly during early-range positions in which elastic tension is low. As the band becomes progressively elongated toward end-range, the resistance increases and muscle activation may become more comparable to that observed with conventional resistance modalities [37]. To offset comparatively low early-range loading, investigators have recommended increasing initial band tension through pre-stretching, reducing resting band length, or combining multiple bands in parallel. These strategies can raise the mechanical demand across a greater proportion of the range of motion without necessarily changing the exercise selected [35].

Activation patterns are also strongly shaped by exercise selection and setup. In core and trunk exercises, elastic resistance can produce erector spinae and abdominal muscle activation comparable to, and in some movement directions greater than, machine-based exercise, while also being perceived as demanding and acceptable by participants [48,49]. In the shoulder complex, elastic resistance exercises can reproduce the high infraspinatus, rotator cuff, and scapular muscle activation commonly targeted in rehabilitation and injury-prevention programs, with activation increasing systematically as band elongation and stiffness progress [44,50,51]. These findings indicate that elastic resistance can be configured to emphasize specific muscles, joint positions, and ranges of motion, provided that the load profile is understood and deliberately manipulated.

However, EMG findings should be interpreted with appropriate caution. Similar EMG amplitudes between elastic and conventional resistance do not necessarily imply identical joint loading, muscle force, mechanical tension, or long-term adaptation. Rather, they indicate that elastic resistance can generate substantial neuromuscular activation when effort, band elongation, and exercise configuration are adequately controlled [52]. This distinction is important in sports medicine and rehabilitation, where the goal is not only to activate a muscle but to prescribe a mechanical and neuromuscular stimulus that is compatible with tissue tolerance, movement quality, injury-prevention goals, functional recovery, and performance progression.

### 4.2. Motor Control, Coordination, and Proprioceptive Demands

Beyond gross activation magnitude, the mechanical characteristics of elastic resistance may impose specific demands on motor control, coordination, and sensorimotor regulation. Because the line of pull is determined by the anchor point rather than by gravity, the user must control a force vector that may act horizontally, diagonally, or rotationally and whose magnitude increases as the band stretches. Mechanically, this configuration may require continuous regulation of movement trajectory, joint alignment, and segmental stability. From a sensorimotor perspective, these characteristics are potentially relevant because functional joint stability depends not only on muscle strength, but also on proprioceptive feedback, neuromuscular coordination, and the ability to generate appropriate motor responses to changing mechanical conditions [44,49,53]. However, these characteristics should be interpreted as plausible sensorimotor demands rather than direct evidence of specific proprioceptive or motor-control adaptations.

Elastic resistance exercises can expose the neuromuscular system to progressive changes in force magnitude and direction within the same movement, potentially requiring continuous adjustments in posture, timing, and intermuscular coordination. This mechanical versatility may partly explain why elastic resistance is frequently incorporated into neuromuscular and proprioceptive training programs, including balance-oriented interventions and rehabilitation protocols targeting dynamic joint control [54]. In patients with chronic ankle instability, a systematic review and meta-analysis reported that elastic-band resistance training and dedicated proprioceptive training both produced improvements in balance and self-reported function [55]. These findings support the use of elastic resistance within interventions targeting functional and balance-related outcomes, but they do not establish that the observed benefits are mediated by a specific proprioceptive or sensorimotor mechanism. This distinction is particularly relevant because chronic ankle instability is characterized not only by mechanical laxity, but also by deficits in postural control, proprioception, neuromuscular activation, and movement confidence [56].

The diagonal and multiplanar patterns readily produced with elastic resistance also align with proprioceptive neuromuscular facilitation principles, in which functional movements are trained across combined planes rather than in isolated single-joint trajectories. By changing anchor position, band orientation, and movement plane, practitioners can approximate selected directional and movement characteristics of sport- or task-specific actions, such as resisted external rotation and abduction in overhead athletes, multiplanar hip control during change-of-direction tasks, or trunk stabilization against rotational perturbations [44,50,51]. Accordingly, elastic resistance provides a practical means of challenging coordination within movement patterns that may resemble aspects of the target activity, although such task similarity should not be interpreted as direct evidence of enhanced proprioception, motor control, or transfer to sport performance.

### 4.3. Joint Loading, Tissue Stress, and Movement-Specific Resistance

The ascending load profile of elastic resistance has direct consequences for joint loading, tissue stress, and movement-specific resistance. Because band tension is typically lower at the beginning of the range of motion and increases as the band elongates, elastic resistance can be configured to modify where mechanical demand is concentrated within a movement. However, band tension should not be interpreted as equivalent to joint loading. The effective load experienced by a joint depends not only on the tensile force generated by the band, but also on the attachment point, limb position, joint angle, movement trajectory, moment arm, and the contribution of body weight or other external loads [21,39]. Musculoskeletal modeling of hip exercises performed with and without elastic resistance has further shown that adding a band can alter muscle forces and joint contact loading, demonstrating that elastic resistance changes the loading environment in ways that may be exploited therapeutically when properly configured [45].

This position-dependent loading is frequently advantageous in rehabilitation, where the goal is to progressively load muscles, tendons, or periarticular tissues while respecting pain, tissue irritability, and healing stage. When anchor position, pre-stretch, and range of motion are carefully selected, elastic resistance can help reduce mechanical demand in sensitive portions of the movement while still providing a meaningful strengthening stimulus in better-tolerated ranges. In knee and shoulder rehabilitation, elastic resistance is commonly used to provide graded loading that can be adjusted according to symptoms, movement quality, and functional progression [50,57]. This capacity to control not only the magnitude but also the location of mechanical stress within the range of motion is particularly relevant after injury or surgery, when repaired or symptomatic structures may require progressive exposure to load rather than uniform or poorly controlled stress across the entire movement.

In performance settings, the same mechanical property can be used with a different objective. When elastic bands are added to barbell exercises, such as the squat, bench press, or deadlift, the ascending resistance profile increases load in the later phase of the lift, where athletes are often mechanically stronger. This can redistribute external demand across the range of motion, reduce the need for deceleration near the end of the concentric action, and increase the requirement for continued force production toward lockout [40,41]. Thus, elastic resistance can be configured either to spare, progressively expose, or overload specific portions of the range, depending on the clinical or performance objective.

The ability to apply resistance in directions that are difficult to achieve with gravity-based loading also expands the range of joints and tissues that can be targeted specifically. Rotational, horizontal, diagonal, and multiplanar loading of the trunk, hip abductors and external rotators, scapular stabilizers, and rotator cuff muscles can be applied directly with bands, often more conveniently than with free weights alone [44,45,50]. This movement-specific loading capacity allows external resistance to be aligned with the demands of a target task or with the mechanics of a structure requiring rehabilitation. Accordingly, the value of elastic resistance lies not only in the magnitude of force it can generate, but in the precision with which its direction, range-specific intensity, and tissue-specific demand can be configured [51].

### 4.4. Rate of Force Development, Power, and Explosive Actions

Many functional and athletic tasks are constrained not only by maximal strength, but also by the rate of force development (RFD), defined as the change in force over a given time interval during the early phase of contraction [58,59]. RFD is strongly influenced by neural drive, motor-unit recruitment, motor-unit firing frequency, and the ability to coordinate rapid force expression within short time windows [58,60]. This quality is particularly relevant in sport actions such as jumping, sprinting, throwing, striking, cutting, and rapid balance recovery, as well as in late-stage rehabilitation and return-to-sport progression, where the capacity to generate force quickly is often as important as maximal force capacity.

Elastic resistance may be useful for targeting rapid force expression because its ascending load profile allows the user to accelerate against relatively low resistance at the beginning of the movement while progressively encountering greater resistance as the band elongates. This configuration can promote high-velocity concentric actions and continued force production through the range of motion [46]. However, this stimulus depends strongly on exercise configuration. If the band is too slack at movement onset, early-range loading may be insufficient to challenge initial force production. Conversely, increasing initial band tension through pre-stretch, selecting a stiffer band, or combining elastic resistance with an external mass can shift the stimulus toward greater force demand while preserving the ascending resistance profile.

Acute studies have shown that adding elastic bands to barbell movements can modify kinetic and kinematic variables such as bar velocity, peak power, force production, and the deceleration pattern near the end of the concentric phase, particularly under appropriate loading conditions and in the later range where band tension is greatest [38,41,61]. Rather than uniformly increasing performance across all conditions, elastic resistance appears to redistribute mechanical demand across the repetition, increasing the requirement for continued acceleration and force production toward end-range. This is especially relevant in exercises such as the squat, bench press, and deadlift, where athletes are often mechanically stronger near lockout and where conventional loading may require deceleration before movement completion [40,41].

Training studies extend these acute observations. High-velocity elastic-band training has been shown to improve knee-flexor strength at high angular velocities and sprint performance comparably to heavy resistance training, while explosive band-based programs have improved power-related and throwing-performance outcomes in team-sport athletes [62,63,64]. These findings position elastic resistance as a practical tool for developing rapid force capabilities, not only maximal strength. In rehabilitation, this may be particularly useful when progressing from isolated strengthening toward faster, task-specific actions; in performance settings, it may support the development of explosive qualities relevant to acceleration, jumping, striking, and change-of-direction tasks.

These observations are consistent with the principle of velocity specificity, whereby adaptations tend to be greatest at or near the velocities at which training is performed [65]. Because elastic resistance can facilitate high-velocity concentric actions against an ascending load, it provides a stimulus aligned with the high-velocity region of the force–velocity spectrum that underpins power development. In contrast, combining elastic resistance with heavier external loads may shift the stimulus toward the higher-force, lower-velocity region of the spectrum [66]. The capacity to bias training toward different neuromuscular qualities by manipulating the resistance configuration, intended movement velocity, and the presence or absence of an accompanying external load makes elastic resistance a flexible tool for targeting maximal strength, RFD, power, or sport-specific explosive actions [46,67].

## 5. Exercise Prescription, Load Monitoring, and Progression

### 5.1. Key Prescription Variables

Effective resistance training depends on the systematic manipulation of acute programming variables, including exercise selection, intensity, volume, frequency, tempo, range of motion, rest intervals, and exercise order, according to the trainee’s goals, training status, and clinical condition [68,69]. These principles apply equally to elastic resistance training, but the variable nature of band tension requires that each prescription variable be interpreted in relation to the mechanical behavior of the device. Exercise selection and set-up define the plane of movement, line of pull, anchoring configuration, and range over which tension is applied, thereby determining which muscles are loaded, where in the range of motion the demand peaks, and how resistance is transferred to the joints involved [21,36,44].

Intensity is the prescription variable that differs most clearly from conventional resistance training. In elastic resistance exercise, the external load is determined by band properties and exercise configuration—including band stiffness, color, width, initial length, pre-stretch, percentage elongation, anchoring distance, range of motion, and the number of bands used in parallel, whereas relative intensity depends on the relationship between this external demand and the individual’s force-generating capacity [9,19]. The level of effort, in turn, reflects how close the performed repetitions are to task failure [70]. Band force can be increased by selecting a stiffer or wider band, adding bands in parallel, increasing pre-stretch, or increasing relative elongation [19,22,71]. Therefore, prescribing only “a red band” or “a medium-resistance band” is insufficient unless the initial length, final elongation, anchoring position, and range of motion are also specified. Elastic resistance should thus be prescribed as a configuration-dependent external load and interpreted relative to the individual’s capacity and proximity to task failure.

Volume can be prescribed conventionally through sets, repetitions, or total time under tension, but its interpretation should consider the load profile imposed across the range of motion. The evidence base includes low-load, high-repetition protocols commonly used in early rehabilitation and clinical populations, as well as heavier or combined elastic loading strategies used for strength and power development [41,72]. Tempo and intended velocity are particularly consequential with elastic resistance because they interact with the ascending load profile. Slow, controlled actions may emphasize movement quality, end-range control, and sustained tension, whereas explosive actions exploit the relatively lower early-range resistance to promote acceleration and power-oriented adaptations [46,63].

Frequency, rest intervals, and exercise order complete the prescription. General resistance-training guidelines recommending two to three sessions per week for most health and fitness goals can be applied to elastic resistance, with frequency adjusted according to recovery capacity, tissue tolerance, symptom response, training status, and rehabilitation stage [68,73]. Rest intervals should be scaled to the intended adaptation, with shorter rest periods generally favoring local muscular endurance and metabolic stress, and longer rest periods favoring strength, power, and movement quality. Exercise order typically progresses from larger multi-joint tasks to smaller single-joint exercises, although the portability and rapid reconfiguration of elastic bands make circuit-based, whole-body, home-based, and field-based formats especially practical [72].

The conventional logic of resistance-training prescription is transferable to elastic resistance provided that intensity and progression are defined in relation to the configured external load and the individual’s capacity.

In children and adolescents, these prescription principles should be applied with particular emphasis on technical competency, appropriate supervision, and gradual progression of training demand. Elastic resistance may be especially practical in youth settings because external resistance can be progressively adjusted through the configuration-dependent strategies described above. However, band color alone should not be used as a surrogate for training intensity. Exercise selection and progression should be individualized according to training experience, physical development, movement competency, and the ability to maintain appropriate technique throughout the prescribed repetitions [2].

### 5.2. Load Quantification and Monitoring

The most persistent practical challenge in elastic resistance training is quantifying the load actually imposed. Color alone is an unreliable indicator of force because resistance levels vary across manufacturers, distributors, band widths, production batches, and states of material wear [8,22]. This challenge is especially consequential in sports medicine and rehabilitation, where underloading may fail to stimulate adaptation, whereas excessive or poorly controlled loading may aggravate symptoms, exceed tissue tolerance, or compromise movement quality.

Two complementary approaches have emerged to address this problem. The first is mechanical quantification. Laboratory characterization of band tension as a function of elongation has produced reference values, regression equations, and loading tables that allow a target force to be translated into a prescribed percentage of elongation for a given band [19,22,26,71]. This approach improves the reproducibility and precision of elastic resistance prescription by moving beyond color-based classification toward quantitatively defined loading parameters. Recent biomechanical work further reinforces that band stiffness (i.e., the band-specific elastic constant) and the degree of elongation are key determinants of the force generated, supporting the need to prescribe and report elastic resistance using mechanical parameters rather than color alone [19].

The second approach is perceptual monitoring. Rating-of-perceived-exertion scales adapted for resistance exercise, including the OMNI-Resistance Exercise Scale, have been validated for use with elastic bands and shown to track exercise intensity in relation to physiological and mechanical indicators such as heart rate and applied force [17]. More recent work has extended perceptual monitoring to elastic-band velocity-based training contexts, suggesting that resistance-specific perceived exertion scales can provide a practical and low-cost strategy for adjusting load when maximal testing or direct force measurement is impractical [16]. Submaximal elastic-band tests have also been proposed to estimate maximal strength in upper- and lower-limb exercises, offering another practical alternative for populations in whom one-repetition maximum testing may be inappropriate or difficult to implement [74].

Mechanical and perceptual approaches should be viewed as complementary rather than competing. Mechanical quantification improves reproducibility by estimating the external force generated by a given band configuration, whereas perceptual monitoring captures the individual’s internal response to that external demand. Used together, they allow elastic resistance to be prescribed, monitored, and adjusted with a level of rigor closer to conventional resistance training while preserving the portability and adaptability that make bands useful in clinical, home-based, field-based, and sport settings.

A minimum reporting standard for elastic resistance interventions should therefore capture device characteristics, mechanical configuration, exercise execution, and the methods used to monitor and progress the training load [8,19,22,26]. To facilitate standardized reporting and improve reproducibility across studies, the specific recommended parameters are summarized in Table 1. Without these details, replication and clinical translation remain limited.

### 5.3. Progression Models Across Rehabilitation and Performance Settings

Progressive overload, defined as the systematic increase in training demand as the trainee adapts, remains the organizing principle of resistance-training progression. General progression models can be operationalized with elastic resistance by manipulating several variables: progressing to a stiffer band, increasing pre-stretch, reducing initial length, increasing anchor distance, lengthening the working range, adding bands in parallel, increasing repetitions or sets, modifying tempo, or increasing intended movement velocity [17,35]. In rehabilitation, these adjustments should be embedded within criteria-based, phase-progressed frameworks in which loading advances according to pain, tissue tolerance, movement quality, strength recovery, functional capacity, and return-to-sport readiness rather than time alone [75,76,77].

A particular strength of elastic resistance is the granularity it affords. Whereas free weights often progress in discrete increments that may be too large for deconditioned, older, painful, or post-operative individuals, band-based load can be adjusted almost continuously through small changes in its mechanical configuration [19,22,71]. This allows small, tolerable steps in overload and makes elastic resistance especially suitable for early rehabilitation, symptom-guided progression, and populations with fluctuating capacity [34,75].

This granularity also aligns with autoregulatory programming. In clinical and athletic settings, the load required to produce a given internal response may vary from session to session according to pain, fatigue, sleep, recovery, psychological readiness, or accumulated training stress. Validated perceived-exertion scales and submaximal elastic-band strength tests allow practitioners to adjust elastic resistance according to the individual’s day-to-day status while maintaining a structured progression model [16,17,74]. Used in this way, progression becomes responsive rather than fixed, which is advantageous in rehabilitation and return-to-sport contexts where both underloading and overloading may delay recovery.

Empirically, elastic resistance programs that apply progressive intensity over sufficient duration tend to produce meaningful improvements in strength, function, balance, and performance across clinical and conditioning contexts [32,78,79]. However, the magnitude of adaptation depends on whether the intervention provides an adequate dose, whether progression is clearly defined, and whether the mechanical characteristics of the band are reported with enough detail to allow replication. Therefore, progression should be described in terms of the total mechanical and perceptual stimulus imposed by the exercise.

### 5.4. Safety, Adherence, and Implementation Considerations

Elastic resistance has a favorable practical safety profile. The absence of a dropped mass, the capacity to perform exercises in supported positions, and the possibility of applying low external loads make bands suitable for deconditioned, painful, older, and post-operative populations when exercises are appropriately selected and supervised [54,78]. Nevertheless, elastic resistance is not risk-free. Practical safety considerations specific to the modality include secure anchoring, inspection for material wear, avoidance of excessive pre-stretch, control of the return phase, and attention to band recoil, especially when bands are used near the face, shoulder, or surgical regions. Although population-based incidence data are currently unavailable, ocular injuries associated with resistance-band recoil have been documented in case reports and clinical case series [80]. Therefore, the true frequency of these events cannot be reliably established from the available literature. Material fatigue, creep, and changes in mechanical properties with repeated use may also alter the actual resistance delivered over time, reinforcing the need for regular inspection and replacement [8].

The reviewed literature did not identify disease-specific contraindications unique to elastic resistance. Accordingly, contraindications and precautions should primarily be determined by the underlying clinical condition, tissue-healing status, symptom behavior, exercise tolerance, balance, and general eligibility for resistance exercise, rather than by the elastic modality itself. In rehabilitation, safety also depends on matching the load configuration to tissue tolerance and symptom behavior. Early-stage exercises may require low pre-stretch, short ranges, supported positions, and slow controlled tempos, whereas later stages may progressively incorporate greater elongation, multiplanar loading, higher velocities, and task-specific resistance directions [44,76,77]. In athletic settings, safety considerations include appropriate anchoring, technical control during explosive movements, and awareness that combining bands with free weights changes the load profile and may alter balance, bar path, or end-range demand [41,81].

Adherence is a decisive determinant of real-world effectiveness. The portability, low cost, and minimal space requirements of elastic devices may reduce logistical barriers by facilitating their use at home, in clinics, gyms, community settings, and field environments. However, sustained adherence also depends on supervision, previous exercise experience, perceived benefits, appropriate progression, and overall program acceptability. Qualitative and mixed-methods evidence suggests that participants generally perceive elastic resistance as acceptable, convenient, and feasible, particularly when exercises are simple and clearly instructed [54,82]. Nonetheless, adherence in unsupervised and home-based settings remains variable, and poor fidelity in exercise configuration or technique may substantially alter the intended exercise dose.

Implementation should therefore combine accessibility with standardization. Written instructions, visual demonstrations, individualized band selection, simple elongation markers, session logs, RPE monitoring, and periodic reassessment can improve fidelity and progression. In telerehabilitation or home-based programs, remote supervision and structured feedback may help ensure that the same exercise prescription produces a consistent and safe training stimulus across sessions. Thus, the practical value of elastic resistance depends not only on its portability but on the clinician’s or practitioner’s ability to prescribe, monitor, progress, and communicate the exercise dose with sufficient precision.

In pediatric and adolescent settings, these precautions should be reinforced through appropriate supervision, secure anchoring, regular inspection of the elastic material, controlled recoil, and avoidance of excessive elongation, particularly when exercises are performed near the face or upper body.

## 6. Elastic Resistance Bands in Rehabilitation and Injury Prevention

### 6.1. Musculoskeletal Rehabilitation and Functional Recovery

Elastic resistance is widely used in musculoskeletal rehabilitation because it provides scalable, portable, and multiplanar loading that can be adapted to pain, tissue tolerance, movement quality, and stage of recovery [83,84]. Unlike fixed external loads, the mechanical demand imposed by an elastic band can be modified through changes in its mechanical configuration and movement execution [19,22,26,71]. This makes elastic resistance particularly useful when clinicians need to progressively expose injured or deconditioned tissues to load while maintaining control over symptoms and exercise technique [76,77]. Comparative evidence also indicates that elastic resistance can produce strength adaptations comparable to conventional resistance when appropriately prescribed, supporting its role as a legitimate loading modality rather than merely a low-intensity adjunct [32].

In knee rehabilitation, elastic-band exercise has been used to improve pain, lower-extremity strength, and function in individuals with knee osteoarthritis and degenerative knee conditions [57,85]. After total knee arthroplasty or total knee replacement, elastic resistance has also been incorporated into rehabilitation programs aimed at restoring strength, mobility, and patient-relevant functional outcomes [86,87]. In these contexts, the main clinical advantage is not simply that elastic resistance is “low load,” but that the load can be finely adjusted and progressed as pain, range of motion, quadriceps activation, gait capacity, and functional tolerance improve.

In the shoulder, elastic resistance forms the basis of many standard strengthening protocols for the rotator cuff and scapular stabilizers. This is particularly relevant because the shoulder requires coordinated force production, dynamic stability, and multiplanar control rather than isolated strength alone. Electromyographic studies show that elastic resistance can reproduce high activation of the infraspinatus, rotator cuff, and scapular musculature targeted in rehabilitation and injury-prevention programs [44,50]. Longitudinal intervention data further indicate that upper-extremity elastic training improves shoulder strength and performance [51].

For spinal and trunk rehabilitation, elastic resistance provides a practical means of loading the trunk in flexion, extension, lateral, and rotational planes. Elastic-band and core exercises can elicit trunk-muscle activation comparable to machine-based alternatives and have been used in programs for chronic low back pain, where the objectives include improving trunk strength, pain, disability, and functional capacity [48,49,88]. The ability to orient resistance horizontally, diagonally, or rotationally is especially relevant for trunk rehabilitation because many functional and sport-specific tasks require multiplanar stabilization rather than isolated sagittal-plane strength.

A distinctive advantage of elastic resistance for functional recovery is the ease with which strengthening can be integrated with neuromuscular and proprioceptive goals. Because the anchor point determines the line of pull, the user must control both the intended movement and the externally applied force vector, which engages stabilizing and synergist musculature alongside the prime mover [44,50]. Accordingly, elastic resistance can be used across the rehabilitation continuum, from early symptom-guided strengthening to advanced sport-specific reconditioning, provided that the exercise configuration, load progression, and clinical objectives are clearly defined. Importantly, improvements in strength, balance, motor control, or functional capacity should not be interpreted as direct evidence of injury prevention. Although these outcomes may modify factors potentially associated with injury risk, demonstration of a preventive effect requires direct evidence of reduced injury incidence.

### 6.2. Injury Prevention and the Return-to-Sport Continuum

Resistance-exercise programs can reduce sports-injury risk, but the preventive effect of elastic resistance as a stand-alone modality is less clearly established. Elastic resistance provides a practical means of delivering targeted strengthening and may be incorporated as one component of multimodal injury-prevention and return-to-sport programs; however, the contribution of elastic resistance itself should not be separated from the effects of the broader intervention unless specifically evaluated [32,89]. Improvements in strength, balance, or neuromuscular function should therefore be considered potential intermediate outcomes rather than direct evidence of reduced injury incidence. Its practical contribution may lie in the capacity to target muscles and movement patterns that are difficult to load with gravity-based resistance alone, including rotator cuff and scapular control, hip abduction and external rotation, trunk rotation, ankle stabilization, and high-velocity limb actions [44,45,49,51,55,63].

In overhead athletes, band-based rotator-cuff and scapular programs are commonly used to develop capacity in musculature exposed to repetitive throwing, swimming, serving, or striking demands. These exercises can reproduce the activation patterns required for external rotation, abduction, and scapular control, making them relevant for both injury-prevention and return-to-sport progressions [44,50,51]. In competitive swimmers, a band-based preventive program has been reported to improve rotator-cuff torque and balance and to limit progressive rotational imbalance across a season, suggesting that elastic resistance may help maintain shoulder capacity under repetitive sport demands [90].

For the lower limb, elastic resistance may contribute to injury-prevention strategies by improving strength, balance, and rapid force expression in muscles relevant to sport-specific risk profiles. High-velocity elastic-band training has increased knee-flexor strength at functionally relevant velocities and improved sprint performance. These outcomes are relevant to hamstring capacity in running and team-sport athletes, although direct evidence for hamstring-injury reduction remains limited [62,91]. In individuals with chronic ankle instability, elastic-band resistance training has been shown to improve balance and self-reported function, with effects comparable to proprioceptive training, supporting its use when the goal is to restore dynamic joint control as well as strength [55].

Elastic resistance also maps well onto contemporary return-to-sport frameworks. Return-to-sport practice is increasingly conceptualized as a criteria-based continuum progressing from return to participation, to return to sport, and ultimately to return to performance, with progression guided by objective milestones rather than time alone [76,77,92]. Within this continuum, elastic resistance can be used early to provide controlled, symptom-guided loading and later to reproduce sport-specific planes, velocities, and coordination demands. This makes it useful for bridging the gap between isolated clinical exercises and the multidirectional, high-speed demands of competition [44,45,64]. However, this compatibility with criteria-based rehabilitation and sport-specific progression should not be interpreted as evidence that elastic resistance independently improves return-to-sport rates, time to return, or successful return-to-performance outcomes, for which direct evidence remains limited.

### 6.3. Aging, Frailty, and Clinical Populations with Functional Limitations

Although the principal focus of this review is sports medicine and rehabilitation, elastic resistance also has relevant applications in older adults and clinical populations with functional limitations. These populations are important because they demonstrate the scalability of the modality across a wide spectrum of physical capacity. In older adults, progressive elastic-band training has been associated with improvements in muscle strength, functional capacity, balance, and body composition, including in populations with sarcopenia or sarcopenic obesity [87,93,94,95]. These findings are consistent with broader resistance-training evidence showing that appropriately dosed strength training improves physical function and muscle-related outcomes in older adults and sarcopenic populations [96,97].

Elastic resistance may contribute to fall-prevention programs by improving modifiable risk factors such as lower-limb strength, balance, and functional mobility, although evidence regarding reductions in actual fall incidence should be considered separately. Meta-analytic evidence supports exercise as a key strategy for reducing falls in older adults, particularly when balance and functional training are included [98]. Band-based programs have reported improvements in chair-stand performance, timed-up-and-go, functional reach, balance confidence, and fear of falling, including in higher-risk groups such as older adults with diabetic peripheral neuropathy [7,72,79,94,99,100,101].

Beyond musculoskeletal and geriatric populations, elastic resistance has been applied in chronic cardiopulmonary, cardiometabolic, and neurological conditions, including chronic obstructive pulmonary disease, hypertension, and stroke [102,103,104,105,106,107]. These applications should be interpreted as complementary to the sports-medicine focus of this review, but they reinforce a broader principle: elastic resistance is useful when exercise needs to be individualized, low-cost, progressive, and feasible in settings where conventional equipment may be unavailable or poorly tolerated.

From a clinical decision-making perspective, elastic resistance may be particularly useful in individuals with low baseline strength, frailty or deconditioning, painful or irritable musculoskeletal conditions, early or intermediate postoperative rehabilitation, and other situations in which small, tolerable, and symptom-guided increments in loading are desirable. Its capacity for gradual adjustment may also be advantageous when exercise tolerance fluctuates or when supported or low-load exercise positions are required [19,22,54,71,75,78]. In contrast, individuals with impaired balance, neurological or cardiopulmonary disease, markedly reduced exercise tolerance, or limited ability to reproduce the prescribed exercise independently may require closer supervision and more conservative progression. Elastic resistance should therefore be selected according to the individual’s functional capacity, symptom response, rehabilitation stage, and supervision needs rather than according to diagnosis alone. When precise application of high absolute external loads is the primary objective, conventional resistance equipment may be more appropriate.

### 6.4. Home-Based, Community-Based, and Telerehabilitation Applications

A practical strength of elastic resistance that is directly relevant to rehabilitation is its suitability for home-based, community-based, and remotely supported delivery. The low cost, portability, and minimal space requirements of bands may reduce logistical barriers to participation in facility-based programs, although adherence also depends on supervision, perceived benefit, exercise experience, progression, and program acceptability [54,82]. Randomized trials show that home- and facility-based band programs can improve strength and physical function, including in pre-frail older adults and long-term-care residents [108,109].

The degree of standardization and supervision required should be considered according to the exercise environment. In supervised clinical or gym-based rehabilitation, practitioners can directly control exercise configuration, movement execution, and load progression. In home-based, community-based, or telerehabilitation settings, the same accessibility is accompanied by a greater risk of between-session variability in these parameters. In these contexts, visual instructions, standardized anchoring references, elongation markers, perceived-exertion monitoring, and periodic reassessment may help preserve training fidelity [16,19,22]. In sport-specific field settings, portability and multidirectional loading are advantageous, but anchoring and technical execution should remain controlled, particularly when higher-velocity or explosive exercises are performed. Thus, ease of implementation should not be interpreted as equivalent to consistency of load reproduction.

Elastic resistance also fits emerging models of telerehabilitation because exercises can be demonstrated, standardized, progressed, and adapted without specialized equipment [82,109]. However, the main limitation in home-based and remotely delivered programs is not the device itself, but the fidelity with which the prescribed dose is reproduced. Small changes in band length, anchor position, elongation, tempo, or range of motion can substantially alter the intended stimulus [19,22,71]. Therefore, home-based elastic resistance programs require clear instructions, visual demonstrations, individualized band selection, safety checks, monitoring of perceived exertion, and simple strategies to standardize elongation and progression [16,19].

Strategies that combine validated perceptual scales with elongation-based prescription may be especially useful in remotely supervised rehabilitation. Perceived-exertion scales provide a practical estimate of the individual’s internal response and level of effort when direct mechanical measurement is not feasible [19,110]. In this sense, the future value of elastic resistance in telerehabilitation will depend less on demonstrating that bands can be effective, and more on ensuring that they can be prescribed, monitored, progressed, and reported with sufficient precision across unsupervised or semi-supervised settings.

These characteristics highlight an important clinical trade-off. Elastic resistance may provide less direct control of absolute external load than conventional weight-based equipment, but it offers substantial advantages in portability, accessibility, multidirectional loading, and adaptability to different exercise positions and environments. Accordingly, the choice of resistance modality should depend on whether precise external-load quantification or practical feasibility and adaptability are the primary requirements of the intervention.

From a practical decision-making perspective, elastic resistance may be preferentially considered when gradual or symptom-guided loading, multidirectional resistance, portability, limited equipment access, or adaptation to home- and community-based environments are priorities. These characteristics may be particularly relevant during early rehabilitation, in frail or deconditioned individuals, in patients with fluctuating exercise tolerance, and when balance, functional movement, or multiplanar control are therapeutic targets. Conversely, free weights or resistance machines may be preferable when precise application of high absolute loads, highly standardized progression, or maximal-strength development is the principal objective. During late-stage rehabilitation and return to sport, elastic resistance may complement rather than replace conventional loading when directional specificity, movement velocity, or field-based exercise implementation is required. Thus, resistance modality should be selected according to the interaction between individual capacity, therapeutic goal, task demands, and exercise environment rather than according to a universal hierarchy of exercise equipment.

## 7. Elastic Resistance Bands in Sport and Performance

### 7.1. Warm-Up and Neuromuscular Preparation

In athletic settings, elastic bands are frequently incorporated into warm-up routines to rehearse task-relevant movement patterns, increase neuromuscular activation, and prepare specific muscle groups for subsequent high-intensity activity. Looped and tubular bands permit targeted loading of muscles such as the glutes [45], rotator cuff [50], and scapular stabilizers [44] in directions that resemble components of later sport actions, while their portability facilitates their use in field-, court-, and competition-based environments. Nevertheless, increased muscle activation during a preparatory exercise should not automatically be interpreted as improved subsequent performance, because the effectiveness of a warm-up also depends on the magnitude of fatigue, task specificity, recovery interval, and characteristics of the athlete [111].

Band-loaded conditioning activities have also been investigated as strategies for eliciting post-activation performance enhancement (PAPE). PAPE and post-activation potentiation (PAP) should not be treated merely as alternative terms, as they are defined by different outcomes. PAPE refers to improvements in subsequent voluntary performance following a conditioning activity, whereas PAP should be reserved for increases in electrically evoked twitch force [112]. Squat-based conditioning activities incorporating elastic resistance have been reported to elicit PAPE, expressed as improvements in subsequent voluntary explosive performance under selected loading and recovery conditions [111]. A proposed mechanical rationale is that the ascending resistance profile may permit substantial force production while maintaining the intent to accelerate through the later portion of the movement.

However, PAPE responses are highly individual and reflect the net influence of fatigue and several performance-enhancing processes, including changes in muscle temperature, muscle water content, and muscle activation; they should not be attributed solely to PAP. Their magnitude depends on the type and intensity of the conditioning activity, the athlete’s strength and training status, the biomechanical similarity between the conditioning activity and the subsequent task, and the recovery interval separating them [111,112]. Moreover, band-based conditioning activities may not consistently outperform a comprehensive, muscle-specific traditional warm-up. Elastic resistance should therefore be considered a flexible means of providing task-specific preparation rather than a universally effective acute performance-enhancement strategy.

From a practical perspective, elastic resistance is best used as a targeted component of a comprehensive warm-up when it reproduces relevant muscles, force directions, or movement patterns, rather than as a universal replacement for traditional warm-up strategies.

### 7.2. Strength, Power, Sprint, and Change-of-Direction Performance

Elastic resistance can contribute to strength and power development both as a stand-alone modality and as a form of variable resistance combined with conventional external loads. These applications should be distinguished because they impose different mechanical and neuromuscular demands. When bands are attached to a barbell during exercises such as the squat, bench press, or deadlift, they redistribute the external resistance across the range of motion by reducing the relative contribution of the elastic load in less mechanically advantageous positions and increasing it as the bands elongate. Depending on the exercise, total load, band contribution, and movement intention, this configuration may promote continued force production toward end-range and reduce the deceleration commonly observed near the completion of the concentric phase [40,41,61,81].

Acute kinetic and kinematic responses should nevertheless be interpreted cautiously, as variable resistance does not consistently increase force, velocity, and power simultaneously across all loading conditions [40,61,113]. These responses are influenced by whether total resistance is matched between conditions, the proportion of the total load provided by the bands, and the specific mechanical variable examined [81,113,114]. They may also vary according to the athlete’s training and strength characteristics and the region of the movement being analyzed [41,114,115]. Accordingly, the mechanical effects of band-assisted barbell training may be more appropriately characterized as a modification and redistribution of loading across the range of motion, often reducing relative resistance in mechanically disadvantaged positions and increasing it as the bands elongate, rather than as a uniform improvement in all performance variables [41,114,115].

Over longer training periods, combined elastic and free-weight resistance has produced meaningful improvements in maximal strength, with some studies reporting greater adaptations than constant resistance alone [71]. A systematic review and meta-analysis similarly reported greater improvements in maximal strength with variable- than with constant-resistance training, although the magnitude of the benefit varied according to training status and intensity [33]. In trained individuals, advantages were more apparent when relatively high loads were used, whereas different loading patterns were observed in untrained participants [33]. These findings suggest that the effects of variable resistance depend on how it is programmed rather than on the presence of bands alone.

As a stand-alone modality, elastic resistance may also improve sport-related physical qualities. Recent evidence has reported positive effects on lower-limb explosive power, sprint performance, and change-of-direction ability in team-sport athletes [6]. Variable-resistance interventions have likewise been associated with improvements in lower-limb explosive performance in athletic populations [42]. Mechanistically, the lower external resistance encountered during the early concentric phase in some variable-resistance configurations may facilitate initial acceleration, whereas the progressive increase in band tension requires continued force production through the later portion of the range of motion. However, insufficient initial band tension may limit the early-range loading stimulus, while an excessive elastic contribution to the total load may cause movement velocity to plateau or decline, thereby constraining power output [41,61,81,113,115]. Power-oriented prescription therefore requires careful manipulation of band stiffness, pre-stretch, range of motion, external load, and intended movement velocity [46,63].

Taken together, the available evidence suggests that both stand-alone elastic resistance and combined variable-resistance approaches can improve selected strength and sport-performance outcomes. However, these findings should be interpreted within the specific loading paradigm investigated and should not be extrapolated directly from one configuration to the other. Nevertheless, the magnitude and specificity of adaptation depend on whether the resistance profile, movement velocity, and training task correspond to the targeted athletic quality [67,116].

### 7.3. Sport-Specific Applications

The directional versatility of elastic resistance facilitates the design of exercises whose force vectors and movement patterns approximate selected components of sport-specific actions. In overhead and throwing sports, bands can be oriented to resist external rotation, internal rotation, abduction, horizontal movements, and diagonal patterns relevant to throwing, serving, striking, and swimming [50,51]. This makes elastic resistance useful for warm-up, supplementary strengthening, injury-prevention programs, and the later stages of return-to-sport preparation.

In handball, structured elastic-resistance programs have improved upper-limb power and throwing-related performance, while explosive band exercises have been used to develop sport-relevant power characteristics [63,64]. Studies in adolescent handball players, distinct band-based interventions—including limb-belt resisted sprinting, contrast elastic resistance, and upper- and lower-limb programs—have reported improvements in selected physical and throwing outcomes [117,118,119]. These findings illustrate the possibility of integrating upper- and lower-limb elastic resistance within the same sport-specific conditioning program.

Applications have also been reported in racket, combat, and other field- and court-based sports. Resistance-band–augmented plyometric training has been associated with improvements in selected neuromuscular outcomes in junior tennis players [120], while broader evidence in team-sport populations suggests potential benefits for sprinting, jumping, and change-of-direction performance [6]. Elastic resistance may be practical in youth and developmental settings because it requires limited space and equipment and allows progressive adjustment of external loading, provided that technique and load progression are adequately supervised.

Movement resemblance alone, however, does not guarantee transfer to sport performance. Transfer depends on the correspondence between training and competition in force direction, movement velocity, contraction type, range of motion, coordination pattern, and temporal demands. Moreover, excessive band tension or an inappropriate attachment point may alter technique rather than reinforce it. Sport-specific band exercises should therefore preserve the essential characteristics of the target action while providing sufficient resistance to stimulate adaptation without substantially distorting movement execution.

### 7.4. Practical Integration into Training Programs

Elastic resistance should be integrated into athletic preparation as a complementary loading strategy rather than regarded as a universal replacement for conventional resistance training [32,33]. Although elastic resistance can produce strength gains comparable to those achieved with conventional resistance across different populations, the available evidence remains heterogeneous, and difficulties in standardizing and precisely controlling training intensity have been reported [32]. In trained athletes, stand-alone bands may be particularly useful for accessory strengthening, movement preparation, or high-velocity tasks when an appropriate resistance can be achieved. In contrast, combined band-and-free-weight configurations may be used specifically to create ascending variable resistance and modify the external loading profile across the range of motion [41,113,115]. Owing to their portability and versatility, elastic bands may also help maintain resistance-training exposure when access to conventional equipment is limited [69,116].

Within a periodized program, elastic resistance can be selected according to the adaptation being targeted. Stand-alone bands may be useful for warm-up, movement preparation, and accessory strengthening, particularly when targeted muscle activation or portable resistance is required [39,40,46]. In contrast, combined band-and-barbell loading may be more appropriate when the aim is to provide ascending variable resistance, modify the external load across the range of motion, or increase the requirement for force production during the later portion of the concentric phase [41,61,81,115]. Consistent with contemporary return-to-sport principles, elastic resistance may also be used during late-stage rehabilitation to progressively manipulate loading direction and movement velocity according to the athlete’s capacity and the mechanical demands of the target activity [92,121].

Applied use in athletic settings should follow the load-quantification and reporting principles outlined in Section 5 and summarized in Table 1. When elastic bands are combined with free weights, practitioners and researchers should additionally report the constant external load, the estimated elastic force at standardized positions within the range of motion, and the proportion of total resistance contributed by the bands at those positions [61,81,113,115]. For stand-alone elastic resistance, the minimum recommended reporting parameters are those specified in Table 1.

Perceived-exertion scales and elongation-based estimates provide practical approaches to monitoring band-based training when direct force measurement is unavailable [18,19]. Movement velocity may provide additional information regarding performance and fatigue, but it should not be interpreted as a direct measure of elastic load because the force generated by the band changes continuously with elongation. Combining mechanical prescription with perceptual and performance-based monitoring offers a more defensible strategy for integrating elastic resistance across the microcycle and throughout the continuum from late-stage rehabilitation to athletic performance.

## 8. Conclusions

Elastic resistance bands represent a versatile resistance-training modality whose defining characteristic is a configuration-dependent force profile. The external resistance generated varies according to the mechanical properties of the band, its elongation, and the exercise configuration. Elastic resistance should therefore not be considered a fixed or inherently low-load stimulus, but rather a mechanically adaptable form of loading that can be configured for different clinical, functional, and performance objectives.

The available evidence indicates that appropriately prescribed elastic resistance can elicit substantial neuromuscular activation and produce meaningful improvements in muscle strength, functional capacity, balance, power, sprint performance, change-of-direction ability, and selected sport-specific outcomes. Its directional versatility and capacity for gradual load adjustment make it particularly useful across the rehabilitation continuum, from early symptom-guided strengthening to advanced multiplanar and high-velocity tasks associated with return to sport. Elastic resistance can also complement conventional resistance training by modifying the distribution of external load across the range of motion, supporting continued force production toward the end-range, and providing a practical option when conventional equipment is unavailable or unsuitable. However, it should not be regarded as a universal replacement for free weights or machines, particularly when the principal objective is the precise application of high absolute loads. Its clinical value therefore depends not only on the magnitude of resistance that can be generated, but also on the balance between load-control precision and the feasibility, accessibility, and adaptability required in the intended setting.

The effectiveness of elastic resistance depends on how the exercise is configured, quantified, monitored, and progressed. Accordingly, elastic resistance should be prescribed and reported using configuration-specific mechanical parameters together with appropriate monitoring strategies.

Despite the breadth of available evidence, important limitations remain. Considerable heterogeneity exists in exercise protocols, intervention duration, participant characteristics, band properties, load quantification, outcome selection, and reporting quality. In addition, consistent with the narrative design of the review, no formal risk-of-bias or methodological-quality assessment of the included evidence was performed, which should be considered when interpreting the conclusions. Evidence supporting improvements in strength and functional performance is more established than evidence demonstrating direct reductions in injury incidence or successful return-to-sport outcomes. Future studies should prioritize standardized descriptions of elastic loading, direct measurement or robust estimation of the external force and joint-level demand, comparisons between different resistance profiles, and adequately powered longitudinal trials examining injury prevention, rehabilitation progression, return to performance, and long-term adherence. Greater methodological standardization will be essential to determine not simply whether elastic resistance is effective, but which configurations, doses, and progression strategies are most appropriate for specific individuals, tissues, movements, and performance goals.

Overall, elastic resistance bands are a configurable loading modality with demonstrated utility for strength and functional training and potential applications across rehabilitation and athletic preparation. They may be particularly useful when gradual or symptom-guided loading, multidirectional resistance, portability, limited equipment access, or implementation in home-, community-, or field-based settings are priorities, including during early rehabilitation and in frail, deconditioned, or otherwise low-capacity individuals. During late-stage rehabilitation and return to sport, elastic resistance may complement conventional loading when directional specificity or movement-specific resistance is required. Conversely, free weights or resistance machines may be preferable when precise application of high absolute loads or highly standardized maximal-strength loading is the primary objective. The value of elastic resistance therefore depends on matching the resistance modality to individual capacity, therapeutic goal, rehabilitation stage, and exercise environment, while claims regarding injury prevention and return-to-sport outcomes require stronger direct evidence.

## Figures and Tables

**Table 1:** Minimum recommended reporting parameters for elastic resistance interventions.

Parameter	Minimum Information to Report
Band/device identification	Manufacturer, model or type, and material when available
Nominal resistance	Band color and/or manufacturer-reported resistance level
Band dimensions	Width or thickness when applicable
Resting length	Initial unstretched length used during the exercise
Pre-stretch	Initial elongation or tension before movement begins
Working elongation	Final elongation and/or percentage elongation during the exercise
Anchoring configuration	Anchor location, attachment point, and orientation relative to the participant
Range of motion	Joint or movement range over which elastic resistance is applied
Resistance configuration	Single or multiple bands; stand-alone elastic resistance or combined with external load
Exercise execution	Movement velocity or tempo when controlled or prescribed
Effort/load monitoring	Rating of perceived exertion (RPE), repetitions in reserve, force–elongation estimates or reference equations, submaximal strength tests, functional/performance-based measures, or other monitoring methods
Progression method	Method used to increase or modify training demand across sessions or intervention phases

## Data Availability

No new data were created or analyzed in this study. Data sharing is not applicable to this article.

## Abbreviations

The following abbreviations are used in this manuscript:

EMG	Electromyography
OMNI-RES	OMNI-Resistance Exercise Scale
PAP	Post-Activation Potentiation
PAPE	Post-Activation Performance Enhancement
RFD	Rate of Force Development
RPE	Rating of Perceived Exertion
